# Resistance Exercise Evokes Changes on Urinary Bladder Function and Morphology in Hypoestrogen Rats

**DOI:** 10.3389/fphys.2019.01605

**Published:** 2020-01-29

**Authors:** Fernanda M. Magaldi, Monise Moreno, Cristiane M. Magaldi, Eduardo M. Cafarchio, Patrik Aronsson, Monica A. Sato, Laura B. M. Maifrino

**Affiliations:** ^1^Laboratório de Análise Morfoquantitativa e Imunoistoquímica, Universidade São Judas Tadeu, São Paulo, Brazil; ^2^Deptartamento Morfologia e Fisiologia, Faculdade de Medicina do ABC, Centro Universitário Saude ABC, Santo André, Brazil; ^3^Department of Pharmacology, Institute of Neuroscience and Physiology, Sahlgrenska Academy, University of Gothenburg, Gothenburg, Sweden

**Keywords:** urinary bladder, physical exercise, ovariectomy, estrogen, morphofunctional analysis

## Abstract

Serum levels of estrogen decrease at climacterium and directly interfere with the urogenital tract. Urinary bladder (UB) is responsive to hormonal changes, especially estrogen. Resistance exercise elicits benefits on severe chronic diseases. Nevertheless, it is still unclear whether the resistance exercise directly affects the UB in ovariectomized (OVx) rats. This study focused on investigating the effects of resistance exercise on UB function and morphology in OVx and control rats. Adult female Wistar rats (∼250–300 g, 14–16 weeks old) [control (*n* = 20) and OVx (*n* = 20)] were divided in the following groups: sedentary (SED), and trained over 1 week (acute), 3 weeks (intermediate), and 10 weeks (chronic). Training was carried out in a ladder, with six bouts in alternate days with 75% of body weight load attached to the tail of the animal. Afterward, the animals were isoflurane anesthetized for evaluation of intravesical pressure (IP) changes upon topical administration of acetylcholine (Ach) and noradrenaline (NE) on the UB. At the end of the experiment, the UB was harvested for histological analysis and stained with hematoxylin–eosin and picrosirius red. Ach increased the IP in both OVx and control rats, whereas NE decreased the IP. However, the acute and intermediate groups showed attenuated responses to Ach and NE, while the chronic groups recovered the responses to Ach and NE close to those observed in SED groups. Acute and intermediate groups also showed decreased thickness of the muscular layer, with a reversal of the process with chronic training. In the OVx groups, the acute training reduced the thickness of the smooth muscle and mucosal layers, whereas chronic training increased it. Urothelium thickness decreased in the OVx SED and acute groups. Collagen type I fibers (CI-F) reduced in OVx SED acute and intermediate groups, while collagen type III fibers (CIII-F) increased in the OVx acute group. In the mucosal layer, the volume density of CFs reduced in OVx rats compared to control groups and chronic training resulted in their recovery. Our data suggest that chronic resistance exercise for 10 weeks reversed the functional and morphological changes caused by hypoestrogenism.

## Introduction

The improvement of public health conditions has increased life expectancy globally. As an effect, a greater number of women enter menopause, which may contribute to lower urinary tract symptoms such as urinary urgency, stress incontinence, increased frequency of daytime, nighttime, and urinary incontinence (Varella et al., 2016). The reduction of serum levels of estrogen at climacterium affects directly the urogenital tract. This is particularly observed in the urinary bladder (UB) and urethra due to the common embryological origin, and also because the epithelium of these tissues is responsive to hormonal changes, especially estrogen (Robinson and Cardozo, 2011).

The loss of bladder compliance may result in several changes due to alterations in the extracellular matrix of the bladder, which has been attributed to increased deposition of collagen fibers in the detrusor muscle (Zhang et al., 2016; Fusco et al., 2018). Collagen and elastin are important components of the UB wall, playing a key role in urinary continence. Accumulation of collagen can reduce the conduction of electrical impulses to the bladder wall and thus decreases UB contractility (Susset and Regnier, 1981; Yoshimura and Chancellor, 2002).

Resistance exercise has beneficial effects on severe chronic diseases, reducing risk factors linked to morbidity and mortality, such as obesity, hypertension, dyslipidemia, type II diabetes, and osteoporosis (Gordon et al., 2009; Roberts et al., 2013; Ciolac and Rodrigues-da-Silva, 2016; MacDonald et al., 2016; Daly, 2017; de Sousa et al., 2017; Mcleod et al., 2019). Despite menopause inducing osteoporosis, insulin resistance, dyslipidemia, and sarcopenia, studies in ovariectomized (OVx) rats, which mimic postmenopausal conditions, have shown that 17β-estradiol replacement combined with strength training simulating squat training (65–75% of 1 RM, 12 repetitions, three times a week) improved plasma glucose levels and adiposity, reduced cholesterol and triglycerides, and improved sarcopenia and osteoporosis (Gomes et al., 2018). However, only training restored plasma insulin (Gomes et al., 2018). In contrast, resistance training in female rats that climbed with weights attached to the tail (4–9 climbs, 8–12 dynamic movements per climb, 3 days a week for 12 weeks) decreased the fat depots (mesenteric and retroperitoneal), lipid profile, and lipid content in the liver, soleus, and tibialis anterior muscles either in OVx or intact rats (Leite et al., 2009).

Mixed urinary incontinence and stress urinary incontinence in older women have been treated with pelvic floor muscle training (Dmochowski et al., 2013; Moore et al., 2013; Dumoulin et al., 2017), which has led to improvement in up to 76% of women with urinary incontinence (Dumoulin et al., 2014). Nevertheless, it is still unknown if the resistance exercise can directly evoke a remodeling of the UB and improve its functional capacity in the period following menopause (Nygaard et al., 2005).

Considering the positive effects of resistance exercise in OVx rats described above and also that during the menopause the UB is susceptible to decrease in estrogen levels, our hypothesis is that resistance exercise can remodel and improve the activity of the UB. Thereby, this study focused on investigating possible functional and morphological changes of the UB in OVx and intact (control) rats maintained sedentary or submitted to resistance exercise for 1, 3, or 10 weeks.

## Materials and Methods

### Animals

Adult female Wistar rats (∼250–300 g, 14–16 weeks old, *n* = 40) supplied by the Animals Care of Universidade São Judas Tadeu were used. The animals were housed in plastic cages in groups of four rats/cage with standard chow pellets and water *ad libitum* in an air-conditioned room (20–24°C). The light–dark cycle of the facility was controlled and established as 12 h each. The humidity of the animal room was maintained at ∼70%. All procedures performed in this study were in accordance with the National Institutes of Health Guide for the Care and Use of Laboratory Animals (NIH Publications No. 8023, revised 1978) and were approved by the Animal Ethics Committee of the Universidade São Judas Tadeu (protocol number 024/2016).

### Resistance Exercise

Moderate resistance exercise was carried out using a 110-cm vertical ladder with 80° slope. At the top of the ladder, there was a box for the resting of the animals between the bouts. The overload used during the training effort was maintained at 75% of the total body weight, and due to that, rats were weighted twice a week in order to calculate and adjust the load necessary for each rat. The loads were attached to the tail of the animal.

Training consisted of six climbs per bout, with 1 min of resting between them, and duration according to the training volume (Hornberger and Farrar, 2004; Lee, 2004), in alternating days (3 days/week). The animals were previously adapted over 1 week, on alternate days without body weight load. During the adaptation period, each rat was submitted to six climbs, with the two first climbs being initiated from the final third of the ladder, two of them from the second third of the ladder, and the last two climbs from the beginning of the ladder, with 1 min interval between each climb, according to the adaptation protocol for resistance exercise proposed by Hornberger and Farrar (2004). Rats were manually stimulated to climb the ladder. No electrical shock was used to induce the climbing.

### Functional Evaluation

The animals were weighed, anesthetized with 2% isoflurane in 100% O_2_, and submitted to cannulation of the femoral artery and vein with polyethylene tubing (PE-50 connected to PE-10, Clay Adams, NJ, United States). The UB was also cannulated for intravesical pressure (IP) measurement. Baseline IP was set at ∼7 mmHg for all animals. After recording the baseline IP, pulsatile pressure (PAP), mean arterial pressure (MAP), and heart rate (HR) for 15 min, topical (*in situ*) administration of noradrenaline (NE) (2.0 μg/ml, 0.1 ml, Sigma–Aldrich, St. Louis, MO, United States), acetylcholine (Ach) (2.0 μg/ml, 0.1 ml, Tocris Bioscience, Bristol, United Kingdom), or saline (0.1 ml) was carried out directly onto the surface of the UB. The physiological parameters were recorded in the PowerLab 16 SP data acquisition system (AD Instruments, Bella Vista, Australia). The percent change in IP (%DIP) was calculated as follows: %ΔIP = [(final IP/initial IP)/initial IP] × 100.

### Histological Analysis

Urinary bladder tissue samples were fixed in 10% formaldehyde for 24 h. Afterward, they were dehydrated, diaphanized, and embedded in paraffin. Two histological sections per animal, non-seriated of 5 μm thickness, were collected and stained in hematoxylin–eosin for thickness analysis of bladder wall layers in a light field microscope (Zeiss-Axiostar Plus) and picrosirius red for analysis of type I and type III collagen fibers by polarized light in the microscope (Zeiss-Axiostar Plus).

### Morphometric and Stereological Analysis

Images of the muscular and mucous layers (50×) and transitional epithelium (200×) of the UB were captured by a Sony video camera coupled to the Zeiss Microscope connected to a computer equipped with software (Axio Vision 4.8, Zeiss) used for quantitative analyses.

For the morphometric analysis, measurements of the thickness of the muscular, mucosa, and transitional epithelial layers of the UB were carried out. The analysis was performed in four fields/slice (0°, 30°, 60°, 90°) per layer.

For stereological analysis, eight fields/slice were evaluated. ImageJ software (NIH, Bethesda, United States) was used to quantify type I (red) and III (green) collagen fibers, where a grid containing 196 points was superimposed on the image of the fields and fiber counting was accomplished.

### Experimental Protocol

The animals were weighed twice a week from the third month of life until their euthanasia. At 6 months of age, 20 animals underwent ovariectomy or were left intact. For ovariectomy, each animal was anesthetized with i.p. xylazine (10 mg/kg of b.w., 23 mg/ml Anasedan^®^, Vetbrands, Paulinia, Brazil) plus ketamine (75 mg/kg of b.w., 100 mg/ml, Cetamin^®^, Vetbrands, Paulinia, Brazil), and placed in ventral decubitus position. After a trichotomy, a laparotomy was carried out and the ovary, ovarian duct, and blood vessels were tied together with a nylon wire (tech-lon 4-0), isolated, and harvested. The animals were suture closed and received a prophylactic i.p. injection of benzylpenicillin benzathine (1 mg/kg, 150,000 IU/ml, Teuto^®^, Anapolis, Brazil).

Two months later, OVx and age-matched intact (control, C) rats were submitted to resistance exercise or maintained sedentary (SED) for 1, 3, or 10 weeks (*n* = 5 rats/group).

At the end of the experiments, the animals received an overdose of i.v. sodium thiopental (170 mg/kg, Cristalia Laboratory, Itapira, Brazil) for later bladder removal and weight.

### Statistical Analysis

A Kolmogorov–Smirnov test for normality was used for verifying the data distribution. Once the results fit to a normal distribution, they were expressed as mean ± SEM. Data were submitted to two-way ANOVA, followed by Tukey’s *post hoc* test. Statistical analysis was conducted using the statistical software package Sigma Stat 3.5. Significance level was set at *P* < 0.05.

Below is shown the study design flowchart:


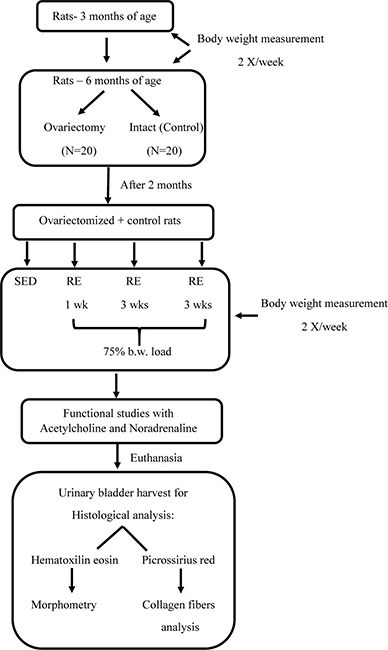


## Results

### Body Weight and Ratio of Urinary Bladder/Total Body Weight in Control and OVx Rats Sedentary and Submitted to Resistance Exercise

The average body weight of the animals increased by 16.75% when weighed at 3 and 6 months, considered adequate due to the development of the animal. However, in OVx animals, a greater increase in body weight was observed at 8 months of age, representing a significant increase of +24.33% compared to age-matched control rats (Table 1).

**TABLE 1:** Body weight at 3, 6, and 8 months (M) of the female rats.

	**3M**	**6M**	**C8M**	**OVx8M**
Body weight	197.7 ± 0.79	236.9 ± 2.93*	249 ± 3.49*	278.9 ± 3.99*^#+^

Data are expressed as mean ± SE. *P < 0.05 vs. 3M; ^#^*P* < 0.05 vs. 6M; ^+^*P* < 0.05 vs. C8M. M, month; C, control; OVx, ovariectomized.

Rats from the control group submitted to 3 or 10 weeks of resistance exercise increased the body weight compared to 1 week of exercise; however, in control rats that underwent 10 weeks of exercise, no further increase in the body weight was observed after 3 weeks of exercise (Table 2). In contrast, OVx rats submitted to 3 and 10 weeks of resistance exercise showed no difference in the body weight along the training period (Table 2).

**TABLE 2:** Body weight of the animals at 1, 3, and 7 weeks of training and urinary bladder (UB) per total body weight (BW) ratio in control and ovariectomized groups.

	**CSED**	**C1**	**C3**	**C10**	**OVxSED**	**OVx1**	**OVx3**	**OVx10**
1 Week	–	164.0 ± 4.0	185 ± 4.2	197.0 ± 2.5	–	262.2 ± 2.7	291.6 ± 10.0	298.6 ± 5.9
3 Weeks	–	–	249 ± 5.3^4^	268 ± 3.6^4^	–	–	286.8 ± 10.6	301.4 ± 3.5
7 Weeks	–	–	–	269.8 ± 3.3^4^	–	–	–	304.6 ± 1.7
UB/BW	0.06 ± 0.007	0.05 ± 0.004	0.04 ± 0.004	0.04 ± 0.004	0.05 ± 0.0	0.08 ± 0.00*^#+⁣&1^	0.08 ± 0.004*^#+⁣&1^	0.02 ± 0.004*^2,3^

Data are expressed as mean ± SEM. *P < 0.05 vs. CSED; ^#^*P* < 0.05 vs. C1; ^+^*P* < 0.05 vs. C3; ^&^*P* < 0.05 vs. C10; ^1^*P* < 0.05 vs. OVxSED; ^2^*P* < 0.05 vs. OVx1; ^3^*P* < 0.05 vs. OVx3, ^4^*P* < 0.05 vs. 1 week. OVx, ovariectomized; UB, urinary bladder; BW, body weight; C, control; SED, sedentary.

Effects of hypoestrogenism in the OVx1 and OVx3 groups were evident when comparing the ratio of UB/total body weight compared to control rats, showing a greater ratio in OVx rats (Table 2).

### Functional Analysis

Baseline MAP of the intact rats was 97 ± 5 mmHg and HR was 297 ± 14 bpm, whereas baseline MAP of OVx rats was 98 ± 3 mmHg and HR was 294 ± 22 bpm.

**FIGURE 1:**
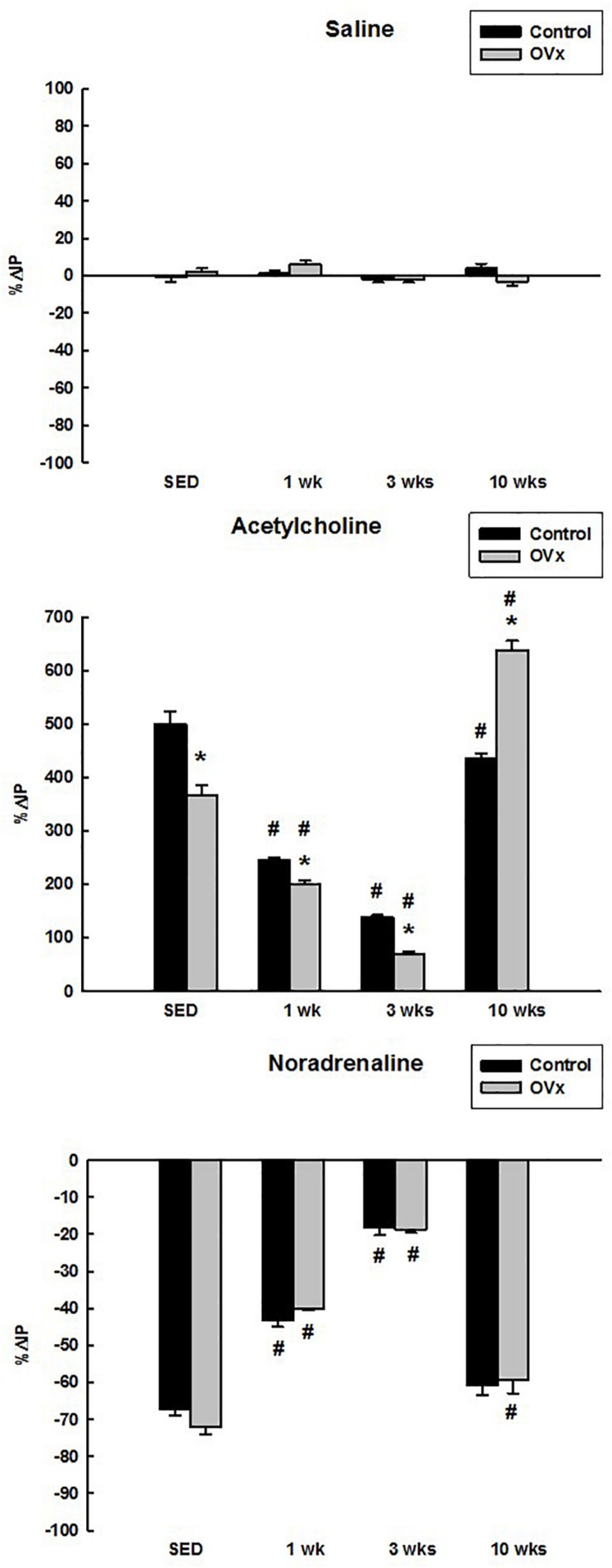
Percent change in intravesical pressure (%ΔIP) evoked by saline, acetylcholine, and noradrenaline in control and ovariectomized (OVx) rats maintained sedentary (SED) or submitted to 1, 3, or 10 weeks of resistance exercise (*n* = 5/group). Data are as mean ± SEM. *P < 0.05 vs. control, ^#^*P* < 0.05 vs. the respective sedentary group.

Saline administrated topically onto the UB elicited no significant change in IP in control (−0.97 ± 2.54% for CSED, 1.42 ± 1.61% for C1, −2.13 ± 1.61% for C3, 4.08 ± 2.33% for C10) and OVx rats (2.20 ± 1.75% for OVxSED, 6.00 ± 1.92% for OVx1, −2.13 ± 1.61% for OVx3, −3.23 ± 2.26% for OVx10) (Figure 1).

Topical (*in situ*) administration of Ach on the UB increased the IP in both control and OVx rats. Nevertheless, the response to Ach was attenuated in OVxSED (366.67 ± 18.00%) compared to CSED (500.00 ± 22.81%) (*P* < 0.001). In addition, the response was significantly attenuated in OVx1 (200.00 ± 7.15%) and OVx3 (68.83 ± 5.58%) compared to OVxSED (*P* < 0.001). Ach induced a greater rise in IP in OVx10 (637.50 ± 17.52%) in comparison to OVxSED and C10 (434.88 ± 10.59%) (*P* < 0.05). Moreover, in control rats submitted to resistance exercise, Ach evoked reduced responses (244.44 ± 6.44% for C1, 138.03 ± 4.29% for C3, and C10) compared to sedentary ones (CSED) (*P* < 0.05). However, the lower responses were observed in C1 and C3 groups in comparison to CSED (*P* < 0.05) (Figure 1).

Noradrenaline *in situ* on the UB decreased the IP in both control and OVx rats with no difference between sedentary rats (−67.12 ± 1.74% CSED vs. −72.16 ± 1.85% OVxSED), exercised for 1 week (−43.33 ± 1.61% C1 vs. −40.00 ± 0.50% OVx1), exercised for 3 weeks (−18.18 ± 2.11% C3 vs. −18.75 ± 0.78% OVx3), and exercised for 10 weeks (−60.87 ± 2.70% C10 vs. −59.20 ± 3.75% OVx10). However, control and OVx rats submitted to 1 and 3 weeks of exercise showed attenuated IP responses to NE compared to the respective sedentary groups (*P* < 0.001). In rats submitted to 10 weeks of exercise, the control group showed similar decrease in IP in comparison to CSED, but not compared to the OVx rats, which still showed a reduced IP response to NE compared to OVxSED (*P* < 0.05) (Figure 1).

No significant changes were observed in MAP and HR after Ach or NE administrated topically onto the UB either in control or OVx rats.

### Morphometric Analysis

#### Thickness of the Muscular Layer

**FIGURE 2:**
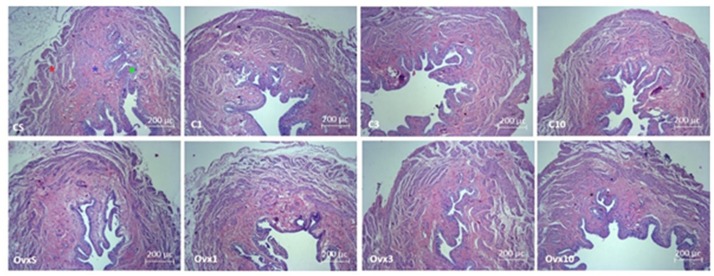
Photomicrograph of the urinary bladder slices in female Wistar rats stained by hematoxylin–eosin technique showing the smooth muscle (red star), mucosa (blue star), and transitional epithelium (urothelium, green star) layers in control and ovariectomized rats maintained sedentary or submitted to 1, 3, and 10 weeks of resistance exercise. C, control; OVx, ovariectomized; S, sedentary.

No significant change in muscle layer thickness was observed in OVx animals (OVxSED, OVx3, and OVx10) when compared to their peers (CSED, C3, and C10), except the OVx1 group, which showed a reduced muscular layer thickness compared to the C1 group (*P* < 0.05) and OVx SED (*P* < 0.05). In the OVx3, the muscular layer was also thinner than in OVxSED (*P* < 0.05). Chronic training (OVx10) was able to reverse this change close to OVxSED thickness (Table 3 and Figure 2).

**TABLE 3:** Thickness of smooth muscle, mucosal, and epithelial layers of urinary bladder in control and ovariectomized rats.

	**CSED**	**C1**	**C3**	**C10**	**OVxSED**	**OVx1**	**OVx3**	**OVx10**
SM	466.9 ± 17.4	487.9 ± 24.3	401.5 ± 19.6^#^	508.2 ± 19.3^+^	507.2 ± 15.7^+^	436.0 ± 22.8^#1^	433.1 ± 20.1^1^	484.1 ± 12.3^+^
Mucosal	437.3 ± 18.6	481.2 ± 27.9	370.5 ± 31.1^#^	460.2 ± 17.2	508.0 ± 16.9*^+^	384.9 ± 30^#1^	462.3 ± 22.4^1^	589.3 ± 21.3*^#+⁣&1,2,3^
Epithelial	39.8 ± 2.4	50.6 ± 3.7*	22.7 ± 1.1*^#^	26.2 ± 1.1*^#^	24.3 ± 0.7*^#^	28 ± 1.1*^#1^	22.1 ± 0.9*^#2^	28.6 ± 0.6*^#1,3^

Data are expressed as mean ± SEM. *P < 0.05 vs. CSED; ^#^*P* < 0.05 vs. C1; ^+^*P* < 0.05 vs. C3; ^&^*P* < 0.05 vs. C10; ^1^*P* < 0.05 vs. OVxSED; ^2^*P* < 0.05 vs. OVx1; ^3^*P* < 0.05 vs. OVx3. SM, smooth muscle; SED, sedentary; C, control; OVx, Ovariectomized.

#### Thickness of Mucosal Layer

Ovariectomy evoked a significant increase in mucosal layer thickness (OVxSED, OVx3, and OVx10) compared to the respective controls (CSED, C3, and C10), except in the acute training group (OVx1), in which the mucosal layer was thinner compared to OVxSED (*P* < 0.05). OVx rats submitted to chronic training (OVx10) showed a significant increase in mucosal thickness when compared to the OVxSED, OVx1, and OVx3 groups (Table 3 and Figure 2).

#### Thickness of the Epithelial Layer

Ovariectomy elicited a significant decrease in the thickness of the epithelial layer in the sedentary (OVxSED) and acute training groups (OVx1) when compared to control (CSED and C1 groups, respectively) (*P* < 0.05). Epithelial layer thickness in groups subjected to intermediate (OVx3) and chronic (OVx10) training was not different compared to controls (C3 and C10). Nevertheless, the OVx10 group showed a thicker epithelial layer compared to OVxSED and OVx3 groups (*P* < 0.05) (Table 3 and Figure 2).

### Stereological Analysis

When analyzing the volume densities of types I and III collagen fibers in the muscular and mucosa layers of the UB wall, a higher prevalence of type I collagen fibers in relation to type III was observed.

#### Volume Density of Collagen Fibers I and III of the Muscular Layer of the Urinary Bladder

**FIGURE 3:**
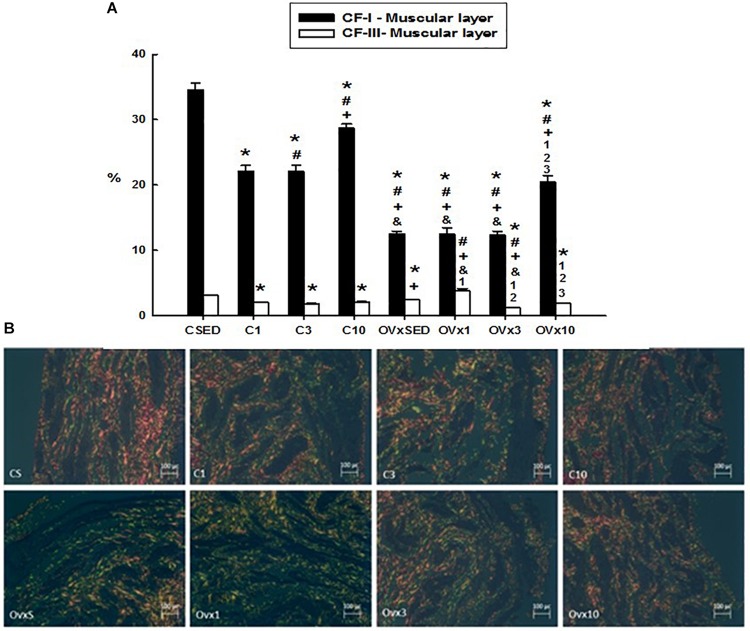
**(A)** Volume density of the collagen type I and III fibers (CI-F and CIII-F) of the smooth muscle layer in control and ovariectomized groups maintained sedentary or submitted to 1, 3, and 10 weeks of resistance exercise. **(B)** Photomicrograph of the urinary bladder smooth muscle layer, showing the collagen type I (red) and III (green) fibers. Polarized Light Microscopy Picrosirius Technique, 200×. C, control; OVx, ovariectomized; SED, sedentary. Data are as mean ± SEM. *P < 0.05 vs. CSED; ^#^*P* < 0.05 vs. C1; ^+^*P* < 0.05 vs. C3; ^&^*P* < 0.05 vs. C10; ^1^*P* < 0.05 vs. OVxSED; ^2^*P* < 0.05 vs. OVx1; ^3^*P* < 0.05 vs. OVx3.

Hypoestrogenism associated with sedentary lifestyle (OVxSED) and acute (OVx1) and intermediate (OVx3) training elicited a significant reduction in type I collagen fibers in comparison to the control rats (CSED, C1, and C3). Chronic training (OVx10) reversed this process close to OVxSED, despite remaining reduced compared to all control groups (*P* < 0.05). When OVx groups were compared to each other, no significant difference was observed in type I collagen fibers among the OVxSED, OVx1, and OVx3 groups (Figure 3).

For the volume density of the type III collagen fibers in the muscular layer, resistance exercise evoked a reduction in the control groups (C1 and C3). For the OVx groups, acute training (OVx1) significantly increased the type III collagen fibers in comparison to the sedentary group (OVxSED), whereas acute and intermediate training (OVx3 and OVx10 groups) showed a significant reduction in these fibers compared to the group exercised for 1 week (OVx1) (Figure 3). In chronic resistance trained rats (for 10 weeks), type III collagen fibers in the muscular layer in OVx rats were reduced in comparison to OVxSED and OVx1 (*P* < 0.05). Chronic resistance trained control rats also showed reduced density of type III collagen fibers compared to CSED (*P* < 0.05).

#### Volume Density of Collagen Fibers I and III in the Mucosal Layer

**FIGURE 4:**
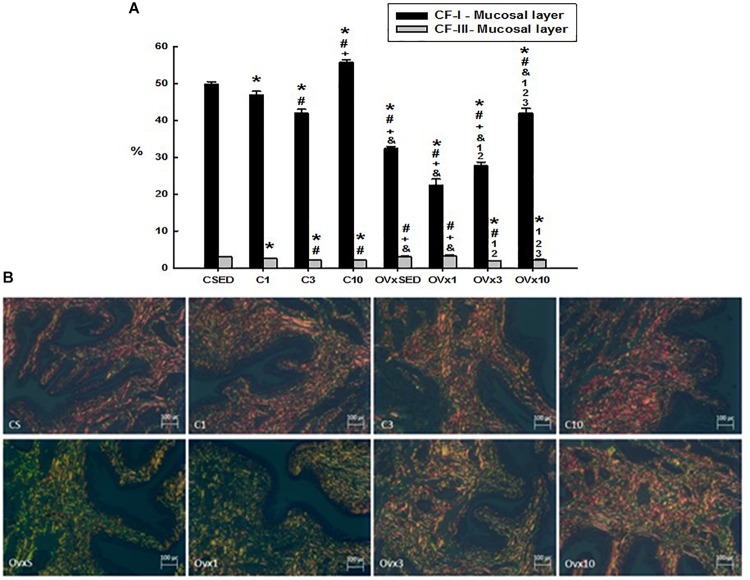
**(A)** Volume density of the collagen type I and III fibers (CI-F and CIII-F) of the mucosal layer in control and ovariectomized groups maintained sedentary or submitted to 1, 3, and 10 weeks of resistance exercise. **(B)** Photomicrograph of the urinary bladder smooth muscle layer, showing the collagen type I (red) and III (green) fibers. Polarized Light Microscopy Picrosirius Technique, 200×. C, control; OVx, ovariectomized; SED, sedentary. Data are as mean ± SEM. *P < 0.05 vs. CSED; ^#^*P* < 0.05 vs. C1; ^+^*P* < 0.05 vs. C3; ^&^*P* < 0.05 vs. C10; ^1^*P* < 0.05 vs. OVxSED; ^2^*P* < 0.05 vs. OVx1; ^3^*P* < 0.05 vs. OVx3.

Ovariectomy significantly reduced the volume density of type I collagen fibers compared to their respective controls (*P* < 0.05). Acute training (OVx1) significantly decreased the type I collagen fibers, whereas chronic training (OVx10) recovered them and even showed a higher density compared to OVxSED (*P* < 0.05) (Figure 4). The control groups submitted to 1 and 3 weeks of resistance exercise also showed a reduced density of type I collagen fibers compared to CSED (*P* < 0.05). In contrast, chronic control trained rats (C10) showed a higher density of type I collagen fibers compared to CSED (*P* < 0.05).

In the control group, the volume of training was closely related to the reduction of volume density of the type III collagen fibers (*P* < 0.05). In OVx groups, neither sedentarism (OVxSED) nor acute training (OVx1) altered the density of type III collagen fibers, but the increase in resistance training (OVx3 and OVx10) promoted a significant reduction of them compared to OVxSED (*P* < 0.05) (Figure 4).

## Discussion

The present study showed that 10 weeks of resistance exercise improved the UB responsiveness to Ach in rats with hypoestrogenism, but not in rats submitted to resistance exercise for 1 and 3 weeks. In addition, hypoestrogenism evoked morphological changes in the bladder wall, reducing the epithelial (urothelium), increasing the mucosal, and eliciting no change in the muscular layer. Chronic resistance exercise for 10 weeks increased the thickness of epithelial and mucosal layers of the bladder wall in hypoestrogenic rats, and reversed the decrease in the muscular layer observed in rats submitted to 1 and 3 weeks of resistance exercise. Conversely, 10 weeks of resistance exercise were unable to recover the type I collagen fibers in the mucosal and muscular layers close to sedentary levels, which were reduced in sedentary and hypoestrogenic rats submitted to 1 and 3 weeks of resistance exercise.

Deprivation of ovarian hormones leads to endocrine and functional disorders, such as dysfunction in the genitourinary tract, loss of libido, osteoporosis, and metabolic cardiovascular diseases, among others (Urtado et al., 2011; Mukaiyama et al., 2015; Gomes et al., 2018; Wang et al., 2018). In the current study, OVx rats showed a significant increase in the body weight, suggesting the influence of hypoestrogenism on this variable. Studies in animals and postmenopausal women (Donato et al., 2006; Marchon et al., 2015) underpin those findings, demonstrating a higher body weight gain in climacteric women with progressive hypoestrogenism that can be also influenced by an inadequate lifestyle.

During the training period, control rats showed a decrease in body weight. In contrast, the loss of body weight in the OVx group was not observed, likely due to hyperphagia, which occurs with estrogen decline (Wade, 1975; Blaustein and Wade, 1976). However, in the current study, food intake measurements were not performed, which is a limitation of this study.

Our findings demonstrated that hypoestrogenism reduced the UB responsiveness to Ach, but not to NE in sedentary rats. Similar data have been observed previously in OVx animals, demonstrating that estrogen plays an important role in the voiding reflexes and castration decreases the cholinergic response (Levin et al., 1980; Yoshimura and De Groat, 1997; Diep and Constantinou, 1999). In the current study, UB responses to Ach and NE in resistance exercised rats for 1 and 3 weeks were greatly attenuated in comparison to sedentary rats, suggesting that either the contraction or the relaxation of the UB were reduced in exercised rats. In OVx rats, the responsiveness of the UB to Ach in resistance exercised rats for 1 and 3 weeks is even lower than in control rats. Despite that, in rats submitted to 10 weeks of resistance exercise, a recovery of the bladder responsiveness to Ach and NE was observed, suggesting a possible functional remodeling in the bladder. In addition, the response of the UB to Ach was enhanced in comparison to OVxSED and C10; thereby, it is possible that the muscarinic receptors underwent upregulation in the membrane of UB cells. In the current study, no labeling of muscarinic receptors was carried out in the UB slices, which is a limitation of this study. Further investigation is still necessary in order to understand the mechanisms involved in our findings. Studies from Zhao et al. (2016) have shown neural structural damage, marked neurodegeneration, and upregulation of the M2 receptors in chronic bladder ischemia (after 8 and 16 weeks of ischemia), which could be implicated in the abnormal detrusor function. In contrast, M3 receptors were upregulated whereas M1 receptors were downregulated after 16 weeks of bladder ischemia (Zhao et al., 2016).

Our data also demonstrated the impact exerted by resistance exercise performed for 1, 3, and 10 weeks on β-adrenergic receptors, directly influencing the relaxation of the bladder. As control and OVx rats, both sedentary or submitted to resistance exercise, showed similar UB responses to NE, the findings are suggestive that hypoestrogenism likely does not influence the up- or downregulation of β-receptors in the bladder. Tyagi et al. (2009) showed that stimulation of the β3 receptor with solabegron, a selective agonist of β3-adrenergic receptors, promotes UB relaxation. The mRNA expression of β1, β2, and β3 adrenoceptors has been demonstrated in both the urothelium and detrusor muscle; thereby, in the current study, it is not possible to consider that only the β3 receptors would be affected by resistance exercise.

The urothelium can be affected by different physiological interventions or stress. Its function is altered with age, presence of mechanical factors as changes in bladder transmural pressure, generation of lateral tension in the urothelium or bladder wall, torsion, movement of visceral organs, and urine composition. In addition, biochemical stress that impacts the urothelial structure and functional activity, including changes in the levels of trophic factors or steroid hormones, should also be considered (Birder and De Groat, 2007; Khanderlwal et al., 2009). The current findings showed a significant increase in the thickness of the epithelial layer (urothelium) of the control group submitted to 1 week of resistance exercise; however, it was noticed that the estrogen deficiency brought a significant decrease of the urothelial layer thickness in the OVx groups (OVxSED and OVx1). Aikawa et al. (2003) have shown that rabbits treated with estradiol resulted in significant urothelial growth and hyperplasia. Thereby, in the present study, two factors possibly affecting the urothelium thickness could be considered, the hormonal changes induced by ovariectomy and mechanical stress due to resistance exercise.

Aikawa et al. (2003), working with hormone replacement, demonstrated that estradiol promoted an increase in smooth muscle layer thickness. Our data showed that hypoestrogenism (CSED and OVxSED) did not significantly change the thickness of muscular and epithelial layers of the bladder. However, the bladder epithelial layer in rats submitted to acute resistance exercise (C1 and OVx1) was increased. The thickness and volume density of the collagen I fibers were reduced in the bladder smooth muscle layer submitted to resistance exercise (OVx1, OVx3, and C3), with subsequent recovery in the groups trained for 10 weeks (C10 and OVx10).

It is known that type III collagen fibers undergo conformational changes to accommodate intravesical volume increase and can be synthesized at the initial repair process (Gonzalez et al., 2016). Therefore, it can be assumed that the acute resistance exercise combined with hypoestrogenism stresses the tissue, leading to an increase of type III collagen fibers, both in the muscular layer and in the mucosa. Other studies have shown an early increase in type III collagen fibers of almost 48% in UB fibrosis (Deveaud et al., 1998). Infiltration of those fibers into the detrusor muscle layer, with subsequent thickening of the perimysial and endomysial connective tissue, suggests an important role of collagen type III in bladder dysfunctions.

The bladder mucosa is also the target of steroid hormones (Saez and Martin, 1981). In the present study, the mucosal layer underwent a significant increase in thickness and a decrease in the volume density of type I collagen fibers in the OVxSED group. However, during acute/intermediate training in both control and OVx groups, a decrease in mucosa thickness and the volume density of type I collagen fibers were observed. Previous studies confirm the relative decrease in collagen I amount in the urethral mucosa of OVx rats, and reversion of the process with estrogen replacement therapy (Rizk et al., 2003; Yang et al., 2009).

In the present study, the mucosal layer showed reduction of type III collagen fibers in the control groups trained for 1 and 3 weeks (C1 and C3) when compared to CSED. Collagen fibers are the main structural elements in the UB and their arrangements in the extracellular matrix allow expansion during filling and, at the same time, avoid excessive expansion in volume capacity. Disarrangement of collagen fibers may alter organ function, resulting in a rigid wall bladder with decreased functional complacency properties (Ewalt et al., 1992).

Further, our data showed that the OVx group exercised for 10 weeks (OVx10) presented a significant increase of the mucosal layer in relation to its control (C10), showing a possible remodeling elicited by the chronic resistance exercise, but with recovery of the type I collagen fibers and reduction of type III fibers. Previous studies of Moalli et al. (2004) have also demonstrated that changes in collagen I and III interfere with the mechanical properties of the organ, since type I is more rigid while type III contributes more to the elastic properties.

## Conclusion

In conclusion, our results suggest that chronic resistance exercise can be beneficial in reducing the negative effects caused by hypoestrogenism in UB activity. The beneficial effects were evident after 10 weeks of training, but not in the less trained animals for 1 or 3 weeks.

Further studies are necessary to elucidate the molecular mechanisms (expression of muscarinic and estrogen receptors in the bladder) involved in the improvement of the functionality of the UB after 10 weeks of resistance exercise in rats with hypoestrogenism, and not at 1 or 3 weeks of resistance exercise. In addition, it is still necessary to understand if an inflammatory process in the UB after 1 and 3 weeks of resistance exercise could be delaying the beneficial effects observed after chronic training.

## Data Availability Statement

The datasets generated for this study are available on request to the corresponding author.

## Ethics Statement

The animal study was reviewed and approved by the Animal Ethics Committee of the Universidade São Judas Tadeu (protocol number 024/2016).

## Author Contributions

FM, MM, and CM trained the animals and carried out all the morphological analysis. EC performed the functional experiments. PA, MS, and LM designed the experiments, analyzed the data, and wrote the manuscript.

## Conflict of Interest

The authors declare that the research was conducted in the absence of any commercial or financial relationships that could be construed as a potential conflict of interest.
